# The Dual Role of Autophagy in Crizotinib-Treated ALK^+^ ALCL: From the Lymphoma Cells Drug Resistance to Their Demise

**DOI:** 10.3390/cells10102517

**Published:** 2021-09-23

**Authors:** Estelle Espinos, Raymond Lai, Sylvie Giuriato

**Affiliations:** 1 CRCT, Inserm UMR1037—Université Toulouse III-Paul Sabatier—CNRS ERL5294, F-31037 Toulouse, France; estelle.espinos@inserm.fr; 2 European Research Initiative on ALK-Related Malignancies (ERIA), Cambridge CB2 0QQ, UK; 3 Department of Laboratory Medicine and Pathology, University of Alberta, Edmonton, AB T6G 2E1, Canada; rlai@ualberta.ca; 4 Department of Oncology, University of Alberta, Edmonton, AB T6G 2R7, Canada

**Keywords:** anaplastic lymphoma kinase (ALK), anaplastic large cell lymphoma (ALCL), stem-like cells, autophagy, crizotinib, targeted therapy, combined therapy, cell survival, cell death

## Abstract

Autophagy has been described as harboring a dual role in cancer development and therapy. Depending on the context, it can exert either pro-survival or pro-death functions. Here, we review what is known about autophagy in crizotinib-treated ALK^+^ ALCL. We first present our main findings on the role and regulation of autophagy in these cells. Then, we provide literature-driven hypotheses that could explain mechanistically the pro-survival properties of autophagy in crizotinib-treated bulk and stem-like ALK^+^ ALCL cells. Finally, we discuss how the potentiation of autophagy, which occurs with combined therapies (ALK and BCL2 or ALK and RAF1 co-inhibition), could convert it from a survival mechanism to a pro-death process.

## 1. Introduction

Anaplastic large cell lymphoma (ALCL) is a rare type of T-cell lymphoma, accounting for approximately 3% of adult non-Hodgkin lymphomas and 10 to 20% of childhood lymphomas [1]. Systemic ALK-positive ALCL (ALK^+^ ALCL), associated with translocation of the *anaplastic lymphoma kinase* (*ALK*) oncogene, has been considered a distinct entity since the WHO revised lymphoma classification in 2016 [2]. Almost 90% of ALK^+^ ALCL in children carry a characteristic t(2;5) (p23;q35) chromosomal translocation, leading to the constitutive activation of the oncogenic fusion protein nucleophosmin (NPM)-ALK, which drives lymphomagenesis through the activation of multiple survival/proliferation pathways [3,4,5]. 

Since the current treatment of this lymphoma (mainly based on aggressive chemotherapy) is not optimal, as 30% of the patients relapse five years post-treatment, considerable efforts have been made to develop therapies directly targeting the NPM-ALK oncoprotein. One compound, the dual ALK/MET tyrosine kinase inhibitor (TKI), was the first-in-class ALK TKI used in clinics for ALK^+^ ALCL [6] and has been shown to be effective in refractory/relapsed cases [7]. However, as reported for other TKIs, escape mechanisms that allow cancer cells to overcome the effects of crizotinib have emerged [8,9]. This led to both the development of a new generation of TKI inhibitors [10,11] as well as a diverse range of combined therapies, in an attempt to preempt relapses and to eradicate the malignant cells [12,13]. In this context, and to improve crizotinib therapy, we investigated the role and regulation of macro-autophagy (hereafter referred to as autophagy) in crizotinib-treated ALK^+^ ALCL [14,15,16,17,18].

Autophagy is a conserved vesicular pathway that allows cells to sequester and degrade either bulk cytoplasm and/or selective substrates [19]. Such unwanted materials are sealed in double-membrane autophagosomes, then submitted to degradation by lytic enzymes. These catabolic reactions occur in autophagolysosomes, which are organelles resulting from the fusion between autophagosomes and lysosomes. This process is tightly orchestrated in five steps, each controlled by different protein complexes. The initiation is governed by the Unc-51-like kinase-1 (ULK1) complex, which is itself regulated by two main kinases, mammalian target of rapamycin complex 1 (mTORC1) (ensuring negative regulation) and 5’ adenosine monophosphate-activated protein kinase (AMPK) (ensuring positive regulation). Then the phosphatidylinositol 3-kinase (PI3K) complex I, including the Beclin 1 (BECN1) protein, controls the nucleation step. The following elongation phase requires the autophagy related 12 (ATG12) and Atg8-family protein conjugation systems, which are mandatory for the lipidation (phosphatidylethanolamine conjugation) of the microtubule-associated protein light chain 3B (MAP1LC3B also known as LC3B) autophagic vacuole classical marker. The process of maturation and fusion with endo/lysosomes is notably controlled by SNARE (soluble N-ethylmaleimide-sensitive factor attachment protein receptor) complexes. Finally, the autophagic cargo is degraded by the lysosomal enzymes and recycled into the cytoplasm. More details on this core autophagy machinery and its regulation can be found in other excellent dedicated reviews [20,21,22,23].

Being such an essential biological pathway for cell homeostasis and integrity, dysregulations of the autophagic process have been implicated in many diseases, including cancers [24]. In this context, autophagy was shown to prevent tumorigenesis in early stages but favor tumor progression in advanced stages [25]. This two-faced role of autophagy is also observed following cancer therapies. Indeed, depending on the treatments and on the types of cancer, evidence for pro-survival or, conversely, pro-death functions of autophagy has been reported [26,27,28,29]. Furthermore, a substantial body of literature points out the possibility of switching from pro-survival towards pro-death autophagy, according to the intensity or the signaling pathways that are stimulated upon anti-cancer treatments. 

**Figure 1:**
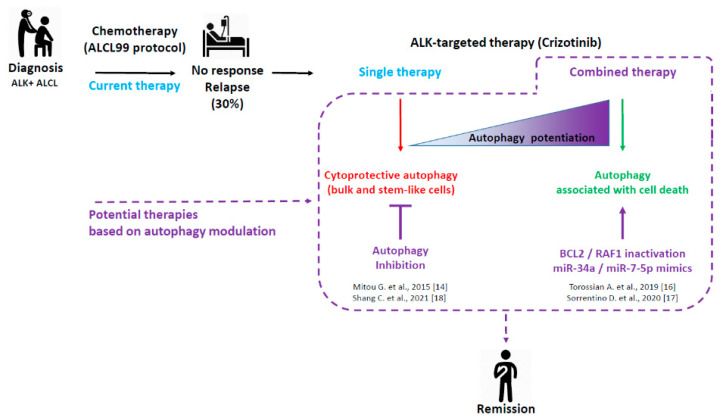
ALK^+^ ALCL treatments: current and potential therapies based on autophagy modulation. Considering the 30% of ALK^+^ ALCL patients who are either refractory to the gold standard ALCL99 protocol or who experienced relapses, research efforts were conducted toward the development of targeted and combined therapies. This scheme summarizes our four fundamental studies, showing the potential therapeutic benefit of modulating autophagy to improve the targeted therapy of ALK^+^ ALCL using crizotinib (dashed lines). Consistent with the known dual role of autophagy (pro-survival or pro-death) according to the therapeutic context, we found that a single treatment of crizotinib led to protective autophagy in bulk and stem-like ALK^+^ ALCL cells, whereas its combination with inhibitors of other key cellular factors triggered an enhanced autophagy associated with cell death.

Over the last few years, our work has focused on the therapeutic modulation of autophagy to improve crizotinib therapy in ALK^+^ ALCL. In this review, we briefly present our previous results, demonstrating either cytoprotective or death-associated autophagy in ALK^+^ ALCL cells, depending on the therapeutic context (Figure 1). Then, we review the literature to highlight the possible molecular mechanisms underlying these autophagy-dependent opposite cell fates. Thus, our review forms the basis for highly interesting investigations in the field of autophagy for the next generation of ALK^+^ ALCL researchers. 

## 2. Pro-Survival versus Pro-Death Autophagy in Crizotinib-Treated ALK^+^ ALCL

### 2.1. Cytoprotective Autophagy in Crizotinib-Treated ALK^+^ ALCL 

#### 2.1.1. Initial Findings

- *Autophagy induction upon ALK inactivation*. We performed a set of experiments to study whether autophagy was induced upon ALK pharmacological or molecular inactivation [14]. We first visualized and quantified an increase in the number of autophagosomes, both by electron microscopy and by LC3B immunohistological or immunofluorescent staining. These results were confirmed by performing classical LC3B turn-over assays and finally by using mRFP-eGFP-LC3 stably expressing ALK^+^ ALCL Karpas-299 cells (generated in our laboratory), which allowed the quantification of the autophagic flux as an increase in the RFP/GFP ratio [30]. Altogether, these methods demonstrated the ability of therapeutically stressed ALK^+^ ALCL cells to mount an autophagic response.
- *Autophagy induction following the inactivation of mTOR signaling*. Mammalian target of rapamycin (mTOR) is an ubiquitously expressed serine/threonine kinase that controls a wide range of key cell functions, including protein synthesis, cell proliferation, and autophagy. The ALK oncogene, acting as a trophic factor, activates mTOR through the MEK/ERK, and to a lesser extent the PI3K/Akt, pathways [31]. As expected, we found that ALK inhibition induced through crizotinib treatment, led to the inactivation of the mTOR pathway, as attested to by the strong reduction in the phosphorylation of the S6 ribosomal protein and 4EBP1 protein, which were both used as read-outs for mTOR activity (unpublished data). In regards to the well-known role of mTOR in the inhibition of the autophagic process, we proposed that mTOR inactivation, as a consequence of ALK inhibition, may account for the activation of the autophagy process [14].
- *Characterization of the cytoprotective function of the crizotinib-induced autophagy*. To decipher whether this crizotinib-induced autophagy affected cell death or cell survival, we tested the cell viability, clonogenic survival, apoptosis, and ability to form xenografted tumors in vivo. We found that upon combined ALK and autophagy pharmacological inhibition, these drugs had a synergistic effect on the reduction of cell viability; they drove cells towards apoptotic/necrotic cell death, strongly reduced ALK^+^ Karpas-299 clonogenic survival, and impaired xenograft tumor growth [14]. Thus, we believe that autophagy is activated upon crizotinib treatment as a stress response, and that its protective function relies on the partial impairment of crizotinib-induced concomitant apoptosis. Indeed, we found, both in vitro and in vivo, that autophagy inhibition potentiated crizotinib-induced apoptosis. These results in ALK^+^ ALCL cells are in line with findings in other cancers and support that the inhibition of cytoprotective autophagy could improve therapeutic outcomes for cancer patients [32,33].

As depicted in Figure 1, we demonstrated in our first study that crizotinib induced autophagy in ALK^+^ ALCL cell lines. Moreover, since autophagy inhibition (either by pharmacological or molecular approaches) potentiated the cytokilling effect of ALK inactivation (either by crizotinib treatment or by an ALK knockdown (KD) approach), our results established that autophagy represented a survival mechanism in therapeutically challenged ALK^+^ ALCL cells.

#### 2.1.2. Bulk and Stem-like Cells in ALK^+^ ALCL

- *Intra-tumoral heterogeneity of ALK^+^ ALCL*. Accumulating evidence supports the existence of intra-tumoral heterogeneity in many types of cancer. In ALK^+^ ALCL cell lines, we found a small but phenotypically distinct cell population that are characterized by their responsiveness to a Sox2 reporter, which we labeled as reporter responsive or RR cells [34]. The bulk cell population is reporter unresponsive and thus labeled RU cells. As the readout for the Sox2 reporter is based on the expression of GFP, RU and RR cells derived from ALK^+^ ALCL cell lines stably transduced with the Sox2 reporter were readily separated and purified using flow cytometry. RR cells consistently showed a higher level of stem-like features, such as chemo-resistance and tumorigenicity. At the molecular level, RR cells are characterized by a high protein level of MYC, as well as evidence of constitutive activation of the Wnt canonical pathway [35]. We have also provided evidence that this RU/RR dichotomy exists in ALK^+^ ALCL tumors, since the MYC expression level detectable by immunostaining is heterogeneous among tumor cells, and its level correlates significantly with that of active β-catenin, a marker of the activated Wnt canonical pathway.
- *Stem-like cells display higher crizotinib-induced autophagic flux*. Using the RU/RR study model, we asked if the cytoprotective effect of crizotinib-induced autophagy is different between the bulk RU cells and stem-like RR cells. To address this question, we first established that the inhibitory concentration of crizotinib at 50% is significantly higher in RR cells than in RU cells (409 nM versus 326 nM). Correlating with this finding, we found that crizotinib triggered a significantly higher autophagic flux in RR cells, as evidenced by some of the assays described in Section 2.1.1. Furthermore, using quantitative RT-PCR, we found that RR cells expressed significantly higher levels of several key autophagy genes, including *ULK1*, *WIPI1,* and *MAP1LC3B*. Importantly, inhibition of autophagy using chloroquine significantly sensitized RR cells to crizotinib, suggesting that autophagy is directly responsible for the cytoprotection against crizotinib. Details of these experiments can be found in reference [18].
- *MYC is a key regulator of the RU/RR dichotomy*. MYC, one of the four inducible pluripotent stem cell (iPS) factors, is known to be frequently deregulated in human cancers. Nonetheless, its role in the pathobiology of ALK^+^ ALCL has not been extensively studied. Our previous studies revealed that the protein level of MYC is a key regulator of the RU/RR phenotype [35]. Thus, overexpression of MYC in RU cells effectively resulted in a gain of Sox2 reporter responsiveness, as well as increased chemoresistance and tumorigenicity (i.e., RR-like). Conversely, knockdown of MYC in RR cells using shRNA or pharmacologic agents resulted in a phenotypic conversion into RU-like cells. With this background, we tested if the different protein levels of MYC between RU and RR cells contributed to their difference in the autophagic flux triggered by crizotinib. This turned out to be the case [18]. Specifically, inhibition of MYC in RR cells significantly dampened the crizotinib-induced autophagic response and its cytoprotective effect. The opposite was observed when RU cells were transduced with a *MYC* expression vector.

Taken together, these results confirmed our initial findings that crizotinib-induced autophagic flux is cytoprotective in ALK^+^ ALCL cells. Furthermore, this autophagy-mediated chemo-resistance is particularly important in protecting stem-like cell populations (Figure 1). Thus, a combination of tyrosine kinase inhibitors and autophagy inhibitors might be an effective approach for eradicating the stem-like cell population in ALK^+^ ALCL.

### 2.2. Autophagy Associated with Cell Death in Crizotinib-Treated ALK^+^ ALCL

#### 2.2.1. Combined ALK and BCL2 Inactivation

- It has been observed for many years, in many studies, that ALK^+^ ALCL has characteristically low expression levels of B-cell Lymphoma 2 (BCL2) proteins [36,37,38], whereas BCL2 overexpression is a classical feature of cancers, including hematopoietic tumors. Our study was the first to report that either crizotinib-mediated inhibition of ALK or its molecular inactivation by specific ALK-targeted siRNA caused an increase in BCL2 levels, highlighting an ALK-dependent BCL2 repression mechanism that is not yet currently elucidated.
- As the BCL2-family proteins are well-known regulators of all major types of cell death, including apoptosis, necroptosis, and autophagy, we next investigated the role of this increase in BCL2 levels on the cellular response to NPM-ALK inactivation or following NPM-ALK downregulation. To address this question, we specifically downregulated BCL2 by RNA interference and showed, as expected, that BCL2 knockdown (BCL2 KD) led to an increase in crizotinib-induced apoptosis, and also to an increase in the autophagic flux [16].
- To decipher whether apoptosis and autophagy were interconnected or occurred independently following combined ALK and BCL2 inactivation in ALK^+^ ALCL cells, we first used the pan-caspase inhibitor Z-VAD-FMK. Although the autophagic flux in the presence of crizotinib was not impaired by the addition of Z-VAD-FMK, we observed that this inhibitor partially rescued cell viability in the siCTL-transfected cells and led to a more pronounced, but still not complete, rescue of cell viability in BCL2 KD cells (unpublished data). Moreover, we observed similar results in response to the inhibition of autophagy using a siRNA targeting the *ULK1* mRNA. All these data led us to propose that the excessive autophagy observed in BCL2-depleted cells drives cell death through multiple modalities, which include apoptosis. This assumption was corroborated by another set of experiments that we performed with a combination of crizotinib and rapamycin (a well-known mTOR inhibitor and strong inducer of autophagy) to amplify the autophagic response without interfering with the BCL2 protein content. We found that the potentiation of autophagy (upon combined treatment) resulted in a strong loss of viability, but with no potentiation of apoptosis, suggesting that another cell death pathway may account for cell killing.
- The mechanistic link between BCL2 inhibition and the overactivation of autophagy is complex. Multiple observations have provided strong support for the prevailing model, in which BCL2 inhibits autophagy through direct interaction with a BH3-like domain of BECN1 [39,40,41]. More recently, Vaux and co-workers [42,43] demonstrated that the inhibition of autophagy by BCL2 is indirect and due to activation of BAX and BAK and thus depends on the presence of an intact intrinsic apoptosis pathway. We therefore performed an array of preliminary experiments to understand which one of these mechanisms could account for the overactivation of autophagy observed in our model. To test the model proposed by Vaux’s group, we first used different agents to interfere with the apoptotis pathway (use of the Z-VAD-FMK compound or use of a siRNA targeting the *BAK* mRNA) and found that inhibiting apoptosis did not impair the potentiation of the autophagic flux observed in cells that received a combined crizonitib/BCL2 KD treatment (unpublished data). We also revealed that, chronologically, autophagic flux induction and potentiation of this flux in response to combined rapamycin/crizotinib treatment is observed prior to the marked decrease in cell viability and the detection of apoptotic cell death, as revealed by Annexin V staining. Altogether, these data suggest a model in which pro-apoptotic effectors (such as BAK) and a functional apoptosis pathway are not a prerequisite for autophagy induction upon crizotinib treatment and autophagy potentiation, either through BCL2 molecular downregulation or rapamycin combined treatment.

Finally, our work provides new insights into the cross-regulation between autophagy and interlinked cell death pathways, including apoptosis in ALK^+^ ALCL. It also provides strong evidence that the molecular targeting of BCL2, combined with crizotinib treatment, could widen the therapeutic options for these patients and potentially improve their outcomes, by reducing the options for cancer cell escape routes (Figure 1).

#### 2.2.2. Combined ALK and RAF1 Inactivation

- Rapidly Accelerated Fibrosarcoma 1 (RAF1) is a key signal transduction protein with serine/threonine kinase activity. A previous study reported that RAF1 downregulation using a RNA interference approach did not impair ALK^+^ ALCL cell growth and proliferation [44], a result in line with emerging literature attributing MEK/ERK independent functions to RAF1 [45,46]. Of interest, RAF1 downregulation in a conditional mouse model for RAF1-induced lung tumorigenesis led to tumor regression associated with an enhanced autophagic flux [47]. Based on this literature, we formulated a hypothesis in which RAF1 could restrain crizotinib-induced autophagy in ALK^+^ ALCL through the serine/threonine inhibitory phosphorylation of a key initiator of autophagy, such as ULK1, as it works for the serine/threonine kinase mTOR.
- To investigate whether RAF1 could phosphorylate ULK1, we first performed specific phospho-serine757 Western blotting on lysates from Karpas-299 cells treated or not with single or combined ALK (using crizotinib) and RAF1 inhibitions (using vemurafenib or a siRNA targeting the *RAF1* mRNA (KD approach)), or using Karpas-299 cells invalidated for *RAF1* expression using the CRISPR/Cas9 system (knockout (KO) approach). Second, we performed in vitro kinase assays using recombinant human RAF1 and ULK1 proteins, in the presence or absence of vemurafenib. Collectively, our results produced evidence supporting the involvement of RAF1 in the inhibitory phosphorylation of ULK1 on its serine 757 residue [17].
- We then evaluated how RAF1 inactivation could affect the autophagic flux, viability, and apoptosis of crizotinib-treated ALK^+^ ALCL cells. We found that RAF1 pharmacological inhibition or molecular downregulation potentiated the crizotinib-induced autophagic flux, which was associated with a loss in cell viability and increased apoptosis [17].

We thus concluded that the combined inhibition of ALK and RAF1 triggered enhanced autophagy, through relieving inhibition of the ULK1 protein, and may represent a more effective therapy, in comparison with single crizotinib treatment, for ALK^+^ ALCL lymphomas (Figure 1). Of note, since vemurafenib is already used in clinics for the treatment of RAF-driven cancers [48] and is efficient for the treatment of mutated BRAF hematological neoplasms [49], such as some multiple myeloma [50,51], hairy cell leukemia [52,53], Langerhans cell histiocytosis [54,55], and Erdheim-Chester disease [55], the clinical translation of our findings may be facilitated.

## 3. Autophagy as a Great Orchestrator of Cell Fate

### 3.1. Molecular Mechanisms Potentially Underlying Pro-Survival Autophagy in Crizotinib-Treated ALK^+^ ALCL

As previously mentioned (see Section 2.1.1), our work demonstrated that ALK inactivation using crizotinib or a siRNA targeting the *ALK* mRNA induced pro-survival autophagy. The possible mechanisms proposed in the literature by which autophagy harbors cytoprotective functions are discussed below.

#### 3.1.1. Impairment of Apoptosis 

Our in vitro and in vivo results indicated that concomitant ALK and autophagy inhibitions led to increased apoptosis [14]. Thus, we proposed that one of the mechanisms accounting for the pro-survival function of autophagy could be the impairment of apoptosis.

- In line with our findings, several studies have highlighted the molecular interplay between autophagy and apoptosis, which provides explanations for the synergy of anti-cancer therapies combining apoptosis-inducing drugs and autophagy inhibitors. As an example, Hou et al. reported the sequestration and degradation of active caspase-8 through autophagy in apoptosis-deficient colon cancer cells submitted to tumor necrosis factor-related apoptosis-inducing ligand (TRAIL). Autophagy inhibition in these settings increased caspase 8 enzymatic activity and promoted apoptotic cell death [56].
- The emerging literature on mitophagy, which is a selective form of autophagy allowing the clearance of defective mitochondria, highlights its role in tumor suppression. Indeed, by limiting the production of pro-apoptotic ROS, mitophagy acts as a survival mechanism, in both growing and therapeutically challenged tumors [57]. This is reminiscent of previous studies showing that impairing macro-autophagy led to defects in mitochondrial homeostasis, thereby increasing ROS levels and DNA damages, and resulting in apoptosis [58,59]. Consequently, inhibiting cytoprotective autophagy/mitophagy in cancer cells leads to oxidative stress-induced apoptotic cell death.
- Recently, a global understanding of how autophagy inhibition could sensitize tumor cells to apoptosis was provided by the group of A. Thorburn [60]. Autophagy, through degradation of the FOXO3a transcription factor, regulates the levels of the pro-apoptotic PUMA protein [61]. A high autophagy activity therefore protects cells by lowering the cellular levels of PUMA, whereas a low autophagy or inhibition of autophagy renders the cells prone to apoptosis. Thus, autophagy may control cancer cell fate by regulating their apoptosis threshold.

Based on this literature, it would be very interesting to study the levels of Caspase 8, FOXO3a, and PUMA proteins and to measure the ROS levels and mitophagy activity in crizotinib-treated ALK^+^ ALCL cell lines.

#### 3.1.2. Autophagy and Cancer Stemness

- *Autophagy is important in the maintenance of cancer stem cells.* Cancer stem cells (CSCs) are known for their propensity to maintain a dormant state and a high level of chemoresistance, which is believed to contribute to treatment failure and disease relapses in cancer patients. The link between cancer stemness and autophagy has been described in a few cancer types [62]. High levels of autophagic flux have been identified in CSCs isolated from cancers of the endometrium, brain, ovary, colon, and breast [63,64,65,66,67]. Importantly, there is accumulating evidence that autophagy plays a crucial role in the maintenance and survival of CSCs. For instance, depletion of Beclin1 in breast CSCs was found to significantly decrease their tumorigenicity in a xenograft mouse model [68]. In another study using ovarian CSCs, the authors found that pharmacologic inhibition of autophagy resulted in decreased chemoresistance, self-renewal ability, and tumorigenicity [65]. In chronic myeloid leukemia, knockdown of ATG4B in CD34-positive stem cell population sensitized these cells to the tyrosine kinase inhibitor imatinib [69]. Similar observations were made in a study using acute myeloid leukemia [70]. As mentioned in Section 2.1.2, inhibition of autophagy using pharmacologic agents (such as chloroquine) and specific siRNA targeting the *ATG7* or *Beclin1* mRNAs in ALK^+^ ALCL significantly lowered the level of crizotinib resistance in the stem-like cell subset.
- *Stem-like cells in ALK^+^ ALCL can be induced.* Autophagic flux can be triggered in response to a diversity of stressful conditions, such as nutrient shortage, hypoxia, chemotherapeutic challenge, and oxidative stress, with the goals of survival and self-preservation. Using the RU/RR study model of ALK^+^ ALCL, we previously found that adverse conditions such as oxidative stress can induce the conversion of RU cells into RR-like cells [71]. Specifically, a small fraction of RU cells treated with hydrogen peroxide acquired Sox2 reporter responsiveness, with a higher level of chemoresistance. Correlating with these phenotypic changes, the MYC protein level substantially increased in these RR-like cells. Although autophagic flux was not assessed in this study of cancer plasticity, it is highly likely that autophagic flux also increased along with the acquisition of cancer stemness. It would be of great interest to examine if autophagy is necessary for this phenotypic conversion in ALK^+^ ALCL. Future experiments will test if the inhibition of autophagy can abrogate the conversion of RU cells into RR-like cells.
- *MYC as a regulator of autophagy and cancer stemness.* While the protein level of MYC is a key regulator of the RU/RR dichotomy in ALK^+^ ALCL, accumulating evidence also suggests that MYC is a regulator of autophagy. As mentioned in Section 2.1.2, modulation of the MYC protein level significantly changed the autophagic response in ALK^+^ ALCL cells. In keeping with our findings, a handful of published studies that directly examined the link between MYC and autophagy shared the same conclusion. One of the first such studies was published by Toh et al. in 2013 [72] and showed that depletion of MYC in HeLa cells impaired autophagosome formation and decreased the level of LC3B-II. The same study also highlighted the importance of JNK1/BCL2 phosphorylation in the regulation of autophagy by MYC. Specifically, MYC promotes the phosphorylation/activation of JNK, which then phosphorylates BCL2 and facilitates the release of Beclin1 from the sequestration of BCL2. A more recent study showed that miR-27b-3p, a microRNA species that is regulated by MYC, can increase autophagy and chemoresistance in colorectal cancer cells via ATG10 [73]. Taken together, we hypothesize that increased autophagic flux induced by adverse conditions promotes cancer stemness and a high protein level of MYC in ALK^+^ ALCL. The resulting high MYC level stimulates further autophagic activity via the JNK/BCL2 and miRNA pathways, thus creating a positive feedback loop.

Based on the presented evidence, we believe that one of the mechanisms by which autophagy provides cytoprotective effects in ALK^+^ ALCL cells is related to its induction/maintenance of cancer stemness, which correlates with a high protein level of MYC. Given the functional versatility of MYC as an oncoprotein, it is likely that autophagy can indirectly stimulate many other cell survival signals and pathways via MYC. We believe that this concept will provided the framework for many interesting future experiments. 

### 3.2. Molecular Mechanisms Potentially Underlying Pro-Death Autophagy in Crizotinib-Treated ALK^+^ ALCL

Although autophagy was primarily identified as a protective mechanism elicited upon ALK-targeted therapy, we found that it was associated with cell death, notably when ALK inactivation was combined with either BCL2 or RAF1 downregulation, which both resulted in a higher autophagic flux [16,17]. The possible mechanisms by which such an enhanced autophagic response could lead to tumor cell death are discussed below. 

#### 3.2.1. Selective Removal of Pro-Survival Substrates or Anti-Apoptotic Factors

We reported in two independent studies that enhanced autophagy in ALK^+^ ALCL cells led to cell death [16,17]. Possible molecular explanations for these findings could be the excessive autophagic degradation of key survival factors or anti-apoptotic factors, which remain to be identified.

- The first autophagic substrate we could consider is the NPM-ALK oncogene itself, since it drives and sustains lymphomagenesis. The hypothesis of NPM-ALK degradation through excessive autophagy is interesting and relevant, since previous reports did indeed show the autophagosomal relocation and degradation of other fusion oncogenes (BCR-ABL, PML-RARA, FLT3-ITD) in different hematological malignancies upon anti-cancer treatment [74,75,76].
- Another interesting substrate, which autophagic degradation was reported to lead to tumor cell death, is the reactive oxygen species (ROS) scavenger catalase [77]. Indeed, such autophagy-mediated ROS accumulation accounts for membrane lipid oxidation, loss of membrane integrity, and subsequent cell demise. Interestingly, ALK^+^ ALCL cells were found to produce a high level of ROS by a pathway involving lipoxygenases (LOX) [78]. Thus, it is tempting to speculate that excessive autophagy by degrading ROS catalase could further increase the ROS content in ALK^+^ ALCL cells until reaching toxic levels responsible for the subsequent cell death. It would be interesting to determine if ROS catalase could be detected in autophagosomes purified from ALK^+^ ALCL cells.
- The same reasoning could apply to the excessive autophagy-mediated degradation of ferritin, which could induce ferroptosis cell death through the accumulation of labile iron and ROS [79,80], as described in erastin-treated MEFs [81]. Thus, the occurrence of autophagy and subsequent degradation of ferroptosis repressors in ALK^+^ ALCL should be investigated. This could be particularly relevant, since ALK^+^ ALCL cell lines and primary tumors have been shown to be resistant to ferroptosis because of the accumulation of squalene, a lipophilic metabolite, in cell membranes and lipid droplets [82].
- Finally, the autophagic degradation of anti-apoptotic proteins, such as Fap-1, which is an inhibitor of Fas-mediated apoptosis (as described in BJAB lymphoma cells [83]), could account for cell death and its occurrence in ALK^+^ ALCL should be investigated.

Therefore, studying whether pro-survival substrates or anti-apoptotic proteins could be detected in autophagosomes and subsequently degraded in ALK^+^ ALCL cells would be interesting. 

#### 3.2.2. Scaffold for Cell Death Complexes

In addition to the selective degradative function of autophagy, it has been described in the literature that autophagosomal membranes may provide a scaffold for different types of cell death complexes, leading, notably, to apoptosis or necroptosis. Of note, in ALK^+^ ALCL, while the occurrence of apoptosis following diverse therapies has been widely reported, the occurrence of necroptosis was only described in CD30 antibody-treated cells in combination with NFκB/proteasome inhibitors [84]. Altogether, these studies confirmed that the apoptosis and necroptosis machinery are functional in ALK^+^ ALCL cells.

- *Scaffold for apoptosis.* Components of the autophagy machinery have been shown to serve as a platform for the apoptotis machinery. As an example, Young et al. demonstrated in MEFs treated with sphingosine kinase inhibitor (SKI) that ATG5- and ATG16L-positive autophagosomal membranes were required to form an efficient intracellular death-inducing signaling complex (iDISC) containing Fas-associated protein with death domain (FADD) and caspase-8 homocomplex [85]. In the same vein, Laussman et al. reported that ATG5 and autophagosome formation contributed to caspase-8 activation following proteasome inhibition in BCL2-overexpressing HeLa cervical cancer cells [86].
- *Scaffold for necroptosis.* A similar scaffold function was described for the necroptosis machinery. Indeed, two studies reported that Obatoclax, an antagonist of BCL2 family proteins, triggers cell death via autophagy through the recruitment of components of the necrosome, such as FADD, RIP1, and RIP3 to autophagosomal membranes [87,88]. Another study demonstrated that Sorafenib, a multi-tyrosine kinase inhibitor, induced autophagy-dependent cell death in ATG5-deficient prostate cancer cells [89]. In this model Sorafenib induced the interaction between p62 and RIPK1, leading to cell death by necroptosis.

In this framework, it is clear that further investigations should be conducted in ALK^+^ ALCL to determine whether apoptosis or necroptosis could occur secondarily to autophagy in crizotinib-treated cells to ensure lymphoma cells’ full eradication, as observed following ALK and BCL2 or ALK and RAF1 combined inactivation. Confirming this assumption, we observed that necrostatin, an inhibitor of necroptosis, partially rescued the cell viability in crizotinib-treated and BCL2-knockdowned ALK^+^ ALCL cells, without impairing the autophagic flux (unpublished data). Thus, as described above, it is tempting to speculate that the autophagy and necroptosis pathways could be interconnected and sequentially activated upon combined ALK and BCL2 inactivation. Furthermore, it would be highly interesting to investigate whether components of the necroptosis pathway could interact with the autophagy machinery in response to crizotinib treatment in ALK and BCL2-KD cells.

#### 3.2.3. Reconstitution of Immune Surveillance 

Autophagy and its role in immunity is a growing field of interest [90,91,92]. As an example, autophagy activation has been shown to participate, in many ways, in anticancer immune surveillance [93]. Regarding ALK^+^ ALCL, some studies reported that NPM-ALK could subvert the immune system, as discussed below. Thus, understanding the role of autophagy in the immunological features of NPM-ALK lymphoma cells is an important research field, holding the promise that autophagy manipulation could improve future ALK^+^ ALCL immune therapies. Below, in the two first bullet points, we remind the reader of the known immune properties of the NPM-ALK oncogene. Then, in the last bullet point, we point out selected studies from the literature reporting immune functions of autophagy, which may be relevant and worth investigating in ALK^+^ ALCL cells.

- *NPM-ALK is an oncoantigen.* Over the last two decades, several publications have demonstrated the immunogenicity of the NPM-ALK oncogene, which can induce in patients, both the production of anti-ALK antibodies [94,95,96], and a T cell immune response against ALK [95,97,98,99,100]. Further validating NPM-ALK as an oncoantigen, the group of Chiarle et al. demonstrated in a mouse model the efficiency of a vaccination therapy using truncated ALK DNA [101].
- *Evidence for NPM-ALK-induced escape from immune surveillance.* Since the NPM-ALK oncogene-elicited immune responses failed to prevent ALK lymphomagenesis, it raised the question of the acquisition of immune escape mechanisms. Indeed, tumor cells can protect themselves from the immune system through several mechanisms, three of these being developed below because of their occurrence in ALK^+^ ALCL cells: (i) by the epigenetic downregulation of CD48 expression and subsequent attenuation of nature killer (NK) cell-mediated cytotoxicity against neoplastic ALK^+^ ALCL cells [102]; (ii) by limiting the presentation of tumor-specific antigen through the downregulation of human leucocyte antigen (HLA) molecules; a recent study reported that the inhibition of the ALK oncogene induced elevated transcript and protein expression of HLA class I, consistent with its increased representation at the cell surface [103], and thus, the ALK oncogene may allow lymphoma cells to evade the immune system by downregulating the expression of HLA class I molecules; (iii) by expressing immunosuppressive factors such as immune checkpoints, which inhibit the activity of tumor-associated T cells. In this framework, NPM-ALK was found to induce the expression of PD-L1 [104,105] through the activation of STAT3 and GRB2/SOS1 signaling networks, and the downstream involvement of the IRF4 and BATF3 transcription factors [104,106]. These important signal transduction studies highlight that NPM-ALK lymphoma can escape immune surveillance and set up the basis for clinical trials using drugs targeting the PD-1/PD-L1 axis. Two recent clinical studies reported the efficacy of anti-PD1 therapy with nivolumab in patients with ALK^+^ ALCL refractory to chemotherapy and ALK inhibitors [107,108]. Altogether, these fundamental and clinical findings support the development of immune-based therapies for controlling the disease.
- *The role of autophagy and autophagosomes in antigen presentation and the immune control of tumors.* Autophagy could likely contribute in many ways to the success of future ALK^+^ ALCL immunotherapies. First, with relevance to the studies showing the NPM-ALK mediated control of HLA class 1 [103] and PD-L1 cell surface expressions [104], autophagy was shown in other settings to facilitate the expression of MHC I molecules [109] and to participate in the selective degradation of the PD-L1 immune-checkpoint [110]. Second, regarding ALK tumor antigen presentation, one can hypothesize that autophagy may potentially participate in NPM-ALK epitope processing and delivery to MHC class I and II molecules. Indeed, several studies reported that the MHC class II loading compartment received cytoplasmic antigen from autophagosomes [111,112] and autophagy was shown in some cases to contribute to the loading of intracellular antigens onto MHC class I molecules [92,113]. It is thus important in this context to investigate whether NPM-ALK could be detected in autophagosomes. Moreover, such potential NPM-ALK containing autophagosomes, once released from dying ALK lymphoma cells, could be captured by dendritic cells in the tumor microenvironment and their content could therefore be redirected to MHC class I complex for processing and cross-presentation, as described previously in other cancers [114]. If so, the development of a NPM-ALK lymphoma autophagosome-based tumor vaccine warrants consideration, as proposed for other tumors [115]. Finally, since autophagosomes are known to contain ATP immunostimulatory molecules, as well as several proteins acting to alarm the immune system [116], autophagy induction in ALK^+^ ALCL cells and the release of their autophagosome content in the tumor microenvironment could potentially contribute to the immunogenic cell death (ICD) of the lymphoma cells. In line with this hypothesis, it is noteworthy that crizotinib treatment in EML4-ALK lung cancer cells was shown to induce the classical features of ICD [117]. Thus, whether these findings are transposable to ALK^+^ ALCL cells is a burning question.

Altogether, these studies demonstrating (i) the identification of NPM-ALK as a potent oncoantigen, (ii) the NPM-ALK-induced escape from immune surveillance, and (iii) the role of autophagy and autophagosomes in tumor antigen presentation and the immune control of tumors strongly advocate for further investigations into how to modulate the autophagic process for the therapeutic success of future ALK^+^ ALCL immunotherapies.

## 4. Conclusions

Autophagy is a fundamental cellular process, controlling cell fate (survival or death) according to the physiological, pathological, and therapeutic context. In the oncology field, many studies have been carried out in different cancer types to understand how to manipulate the autophagic process to improve treatments. In the era of precision medicine, it is likely that including the assessment of autophagic activity (through genome sequencing and autophagy-related gene expression profiling), both in tumor cells and in their microenvironment, at diagnosis and/or following therapy, would lead to the identification of new molecular biomarkers and actionable biologic pathways, which would potentially improve the clinical management of cancers. Of particular note, the recent advances in cancer genomics are now entering the precision medicine management of lymphoid malignancies to provide the best clinical outcomes in patients [118,119,120].

Regarding ALK^+^ ALCL, the therapy has evolved over the years with the noble aim of improving patient survival and quality of life [13]. In addition to the known chemo- and targeted-therapies, immunotherapies are now studied as part of the drug arsenal against ALK^+^ lymphoma. 

Our studies in the field of ALK-targeted therapy demonstrated that “restrained” autophagy (upon crizotinib single treatment) was cytoprotective in bulk and stem-like cells, whereas “enhanced” autophagy (upon combined therapies and additional signaling changes) was associated with cell death. Thus, our work indicated that manipulating autophagy could improve the efficiency of crizotinib-targeted therapy. Table 1 below summarizes the main results regarding autophagy as a target for ALK^+^ ALCL therapy. Of note, studies of combined therapies, targeting ALK and mTOR (which is an upstream negative regulator of the autophagic process) are also included in this table, even if the autophagic flux and its impact on cell fate was not investigated (NI).

Finally, to follow the current burst in cancer immunotherapies, it is of utmost importance to study the role of autophagy in ALK lymphoma cells immunogenicity, immune surveillance, and response to immunotherapies. Indeed, it is likely that, together with the ongoing development of autophagy-modulating drugs [22,124,125,126], immune-checkpoint inhibitors [127,128], and ALK immunotherapies [107,108,129,130], this might lead in the near future to the better management of ALK^+^ ALCL patients.

## Figures and Tables

**Table 1:** Therapeutic modulation of autophagy in ALK^+^ ALCL.

Therapeutic Agent(s)	Target in the Autophagic Process	Autophagy Function	Study Model	References
Crizotinib + Chloroquine	Autolysosomal degradation	Pro-survival	ALK^+^ ALCL cells Xenografted mice ALK^+^ ALCL cells Stem-like ALK^+^ ALCL	[14,18]
Crizotinib + BCL2 KD	BECN1/BCL2 complex *(not yet demonstrated in* *ALK*^+^ *ALCL)*	Pro-death	ALK^+^ ALCL cells Xenografted mice	[16]
Crizotinib + RAF1 KD/KO or Crizotinib + Vemurafenib	ULK1 phosphorylation	Pro-death	ALK^+^ ALCL cells	[17]
Crizotinib + Temsirolimus	mTOR	NI	ALK^+^ ALCL cells Xenografted mice	[121]
Crizotinib + Everolimus	mTOR	NI	ALK^+^ ALCL cells	[122]
Alectinib + Everolimus	mTOR	NI	ALK^+^ ALCL cells	[123]
